# SGLT2 Inhibitors and Uric Acid Homeostasis

**DOI:** 10.3390/gucdd2020014

**Published:** 2024-05-31

**Authors:** Ava M. Zapf, Owen M. Woodward

**Affiliations:** Department of Physiology, University of Maryland School of Medicine, Baltimore, MD 21201, USA

**Keywords:** uric acid, urate, kidney, glucose, SGLT2, gout, SGLT2 inhibitors

## Abstract

A relationship between metabolic disorders and hyperuricemia is well established. The nature of the relationship—risk factor, causal agent, or byproduct—remains unclear. Recent studies of sodium–glucose transporter 2 inhibitors (SGLT2i’s) have established that this pharmacological intervention is beneficial to patients with hyperglycemia and type 2 diabetes mellitus (T2D) and also against the common cardio and renal comorbidities associated with diabetes. Hyperuricemia, or high plasma uric acid levels, is one of the comorbidities mitigated with SGLT2i treatment, raising the potential for using SGLT2i’s as part of the treatment for gout and hyperuricemia. However, the mechanisms underlying the lower plasma urate levels and increased uricosuria produced with SGLT2i’s remains poorly understood. Here, we review the renal physiology of glucose and uric acid transport, the renal consequences of hyperglycosuria and diabetes, the benefits and physiology of SGLT2i use, and discuss several potential mechanisms that may be responsible for the favorable uricosuric effect observed in those treated with SGLT2i’s.

## 1. Introduction

Diabetes mellitus is a major health crisis affecting over 37 million individuals in the United States, with 90% of patients having type 2 diabetes mellitus (T2D), which is characterized by reduced insulin production and insulin resistance [1]. According to the Centers for Disease Control and Prevention (CDC), there are approximately 1.4 million new cases of diabetes per year, with an increasing incidence correlated with older age [2]. People who have a high body mass index (BMI) associated with a lack of exercise and poor diet are at most risk for developing T2D [3]. Many comorbidities may accompany T2D, such as diabetic nephropathy, chronic kidney disease (CKD), hypertension and cardiovascular disorders, dyslipidemia, hyperuricemia, and non-alcoholic fatty liver disease [3,4]. The most common pharmaceutical intervention of controlling hyperglycemia in T2D patients involves glucose-lowering drugs that target the insulin production in the pancreas directly or indirectly (i.e., glucagon-like peptide agonists) [4]. A new class of blood glucose-lowering pharmaceuticals that target glucose reabsorption by the kidney, sodium–glucose transporter 2 inhibitors (SGLT2is), overcome the unwanted side effects of glucose-lowering drugs and provide significant health benefits. These include improved cardiovascular and renal functions, an improved lipid profile, weight loss, and uric acid (primarily urate at physiological pH)-lowering effects [4–6]. The unexpected increase in urate excretion resulting from SGLT2i use suggests a significant potential benefit to those with hyperuricemia and urate pathologies [7] and highlights the need to better understand the well-known but unresolved relationship between urate levels and metabolic disorders, mainly in T2D.

Elevated serum urate, or hyperuricemia, affects an estimated 47 million adults in the United States [8]. Hyperuricemia is significantly more prevalent in men and is the primary cause of gout, the most common form of inflammatory arthritis, with a prevalence of 5.1% in US adults [8,9]. Hyperuricemia increases the probability of urate precipitation and crystal deposition in the synovial fluid of joints, causing gout, or in the kidneys or urinary system, causing nephrolithiasis [8]. Gout is an exceptionally painful form of arthritis and can lead to secondary skeletal malformations and increase the risk of cardiovascular disease [10]. Urate-lowering therapy (ULT) is a critical component in treating gout patients and includes xanthine oxidase inhibitors (Allopurinol and Febuxostat), drugs that block the upstream enzyme responsible for uric acid formation [11]. Increased urate excretion can also be promoted pharmacologically with URAT1 inhibitors (Probenecid, Lesinurad, and Verinurad) in combination with xanthine oxidase inhibitors. Although both types of treatment are effective at lowering plasma urate levels, the increased cardiometabolic risk associated with gout is not mitigated and remains a major clinical concern [12,13]. An unexpected benefit of the new SGLT2i class of drugs is the reduction of plasma urate levels; could lowering urate levels with SGLT2is contribute to the observed reduction in cardiometabolic pathologies? Here, we review the molecular mechanisms and renal physiology of urate and glucose transport, the well-known but mechanistically unresolved association of hyperuricemia and T2D, and how the use of SGLT2is may be beneficial to patients with hyperuricemia and gouty arthritis.

## 2. Glucose and Uric Acid Handling in the Kidney

### 2.1. Uric Acid/Urate

Uric acid is the terminal metabolite of purine metabolism in humans and apes and is a weak organic acid with a pKa of 5.75, existing predominantly as urate (protonated) at physiological pH [8]. The formation of uric acid is catalyzed by an enzyme cascade consisting of xanthine oxidase and xanthine dehydrogenase from the intermediates xanthine and hypoxanthine, respectively [8]. In most organisms, uric acid is converted into the highly soluble byproduct allotonin by the enzyme uric acid oxidase (uricase, *UOX*) [8]. However, the accumulation of three separate mutations in *UOX* has rendered it as a pseudogene (no protein product produced) in humans, strongly suggesting that loss of the uricase enzyme is somehow beneficial or adaptive. The loss of the uricase enzyme function results in conjunction with elevated plasma urate levels that are 5 to 6 times higher in humans than in other mammals [8].

The evolutionary benefit of high uric acid levels or *UOX* loss remains controversial. Supported benefits include its roles as an antioxidant and positive modulator of metabolic pathways during times of starvation [14]. However, distinct disadvantages of elevated plasma urate levels are also present. Elevated plasma urate levels above 6.8 mg/dL, the clinical definition of hyperuricemia, increases the risk of monosodium uric acid crystal precipitation, causing kidney stones and gout [8]. Hyperuricemia is also correlated with multiple comorbidities, including chronic kidney disease (CKD), cardiovascular disease, hypertension, and metabolic disorders, mainly T2D [8].

Circulating plasma urate levels are a balance of urate produced by the liver and through excretion by urate transporters in the kidneys (70%) and intestines (30%) [8]. The first urate transporter identified was URAT1 (*SLC22A12*), discovered using a candidate-based approach, where families with significant plasma uric acid elevations were examined [8,15]. Investigations into other organic anion transporters (OATs) of the *SLC22* gene family followed the URAT1 discovery, where a low affinity for uric acid was indeed determined; however, the involvement of these OATs in maintaining physiological urate homeostasis was inconclusive [8]. Due to the strong heritable component of hyperuricemia and gout, many genome-wide association studies (GWAS) were conducted in an effort to discover novel urate-associated genes [8]. Of the loci discovered using GWAS, three transporter genes, *ABCG2*, *SLC22A12*, and *SLC2A9*, were responsible for the largest portion of measured variability in plasma uric acid levels [16,17]. Functional studies confirmed ABCG2 [18,19] and SLC2A9 [20] as high-capacity and physiologically important urate transporters expressed in the renal tubule and many other tissues [21].

**Figure 1.**
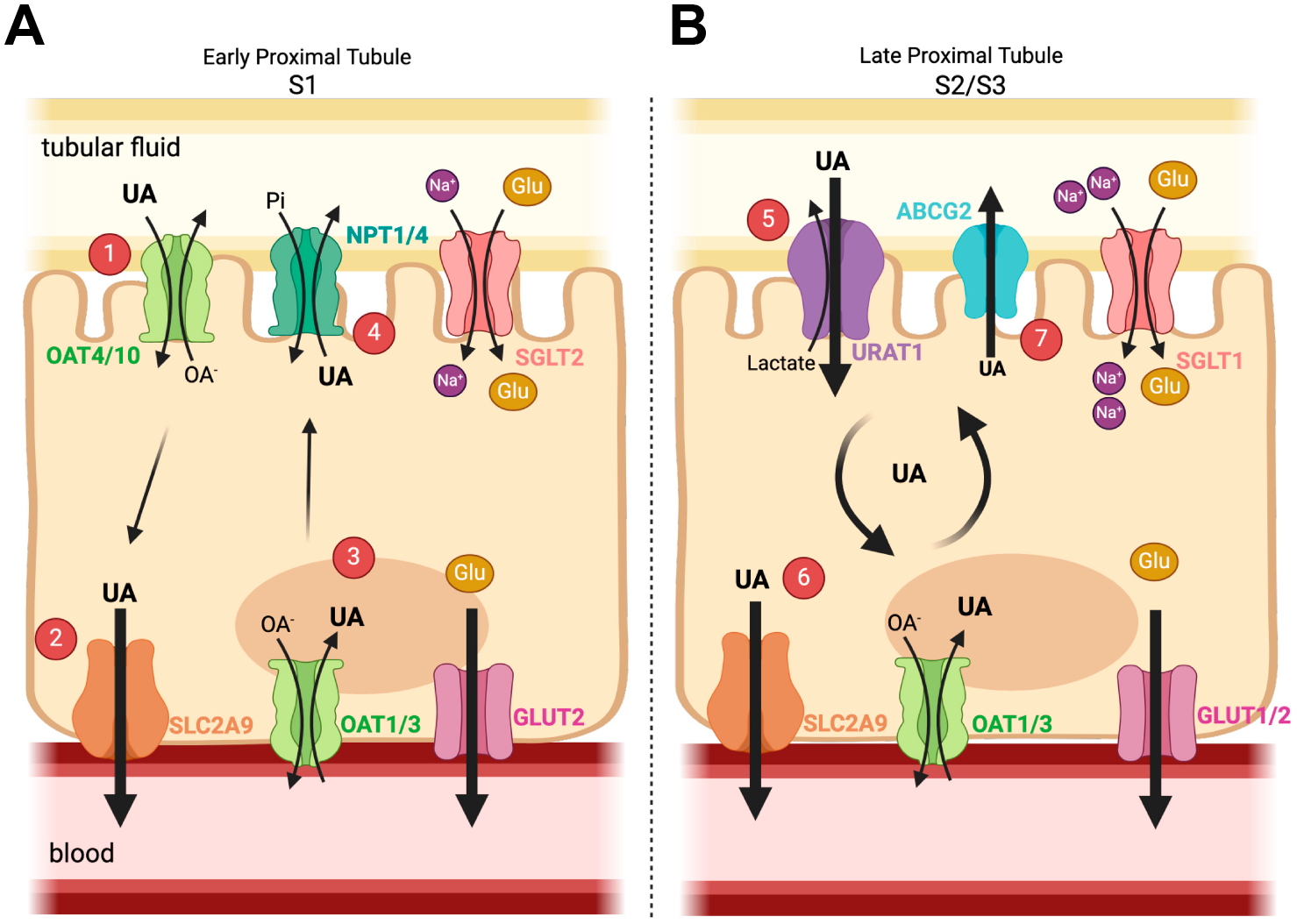
Renal uric acid and glucose transport physiology. (**A**) After being freely filtered by the glomerulus, (1) uric acid is reabsorbed from the tubular fluid by apically expressed OAT4 and OAT10 and (2) reabsorbed back into the blood by basolateral SLC2A9. (3) OAT1 and OAT3 at the basolateral membrane are responsible for transporting uric acid from the blood into the cell for (4) re-secretion into the tubular fluid by apical NPT1 and NPT4. The apical high-capacity/low-affinity glucose transporter SGLT2 reabsorbs approximately 90% of the filtered glucose within the early proximal tubule along with one sodium ion per glucose molecule, where glucose is reabsorbed into the blood by basolateral GLUT2. (**B**) The late proximal tubule (S3 segment) is the chief site of uric acid reabsorption. The majority of the freely filtered uric acid is (5) reabsorbed from the tubular fluid in exchange for lactate by the apical transporter URAT1 and is (6) reabsorbed back into the blood by basolateral expressed SLC2A9. Basolateral OAT1 and OAT3 reabsorb uric acid from the blood back into the cell for (7) re-secretion into the tubular fluid through the specific uric acid secretory transporter ABCG2. The low-capacity/high-affinity glucose transporter SGLT1 is responsible for reabsorbing the remaining <10% of glucose from the tubular fluid using two sodium ions per glucose molecule and is similarly reabsorbed into the blood by basolateral GLUT1/2. Abbreviations: OA^−^ = organic anions; Pi = inorganic phosphate; UA = uric acid; OAT = organic anion transporter; NPT = sodium/phosphate transporter. Images were created using Biorender.com.

The kidney is responsible for the majority of urate excretion; however, renal handling of urate is not straightforward (Figure 1). Freely filtered by the glomerulus, approximately 90% of filtered urate is immediately reabsorbed by the first (S1) segment of the kidney proximal tubule, which is mediated chiefly by bulk organic anion transporters on the apical membrane: organic anion transporters OAT4 and 10 (*SLC22A11/13*) (Figure 1A) [8,22,23]. Urate leaves the cell across the basolateral membrane primarily via SLC2A9 (or Glut9, *SLC2A9*) (Figure 1A) [8,20]. The biological understanding of SLC2A9 function is complex and not completely reconciled. Two splice variants exist in both humans and mice, where the dominant SLC2A9a (long isoform) is expressed at the basolateral membrane along the entire proximal tubule, and SLC2A9b (short isoform) is possibly expressed at low levels apically within the distal nephron [24].

The subsequent downstream segments of the proximal tubule (S2/S3) are responsible for both secreting urate (up to 50% of the original filtered load) and secondary reabsorption (40% of filtered load), resulting in a final fractional excretion of urate of around 10% [25–27]. In the latter proximal segments, the energetic secretion of urate involves basolateral OAT1/OAT3 (*SLC22A6/8*) and apical ABCG2 working together, with reabsorption occurring potentially in the same cells via the apical URAT1 and basolateral SLC2A9 [8,21] (Figure 1B).

### 2.2. Renal Glucose Physiology

Glucose is the primary energetic cellular substrate used to provide necessary energy and is kept within a narrow physiological plasma range of 4–12 mM [28]. The kidney contributes to glucose homeostasis by reabsorbing all the glucose filtered by the glomerulus (~180 g/day) and creating glucose by gluconeogenesis during the fasted state [28]. Under normal conditions (Figure 1), 100% of filtered glucose is reabsorbed in the proximal tubule, a highly energetic tubule segment responsible for the majority of all nutrient, electrolyte, and water reabsorption [29]. Though the proximal tubule requires significant energy to facilitate this bulk reabsorption, the majority of the glucose reabsorbed is transported back into circulation, with renal epithelial cells instead relying on amino acid and fatty acid metabolism for ATP synthesis [5]. Proximal tubule glucose reabsorption occurs at two relative positions along the proximal tubule, regulated by the expression and localization of the two primary apical glucose transporters, sodium glucose transporters 1 and 2 (SGLT1 and SGLT2, coded by the genes *SLC5A1* and *SLC5A2*, respectively) [28]. Freely filtered glucose first reaches the apical SGLT2 transporter, localized in the S1/S2 segments of the proximal tubule [29] (Figure 1A). SGLT2 is a high-capacity, low-affinity glucose transporter that uses the driving force of one sodium ion moving down its electrochemical gradient into the cell to facilitate the transport of one glucose molecule [29], reabsorbing approximately 97% of the filtered glucose load [29]. The basolateral transport of glucose out of the cell and back into the peritubular capillaries occurs via the transporter Glut2 (*SLC2A2*) transporting glucose down a strong chemical gradient (Figure 1A). The remaining 3% of filtered glucose travels down the proximal tubule to the S3 segment, the primary localization of the apical SGLT1 transporter [28]. SGLT1 is a low-capacity, high-affinity glucose transporter that couples the increased driving force of two sodium ions to transport one molecule of glucose [29] (Figure 1B). In the S3 segment, basolateral glucose transport occurs via either Glut1 (*SLC2A1*) or Glut2 down its chemical gradient into the peritubular capillaries [29] (Figure 1B). Loss-of-function mutations in SGLT1 and SGLT2 illuminated their respective roles in renal glucose homeostasis and demonstrated a pivotal, renal-specific role for SGLT2. SGLT2 loss-of-function mutations result in urinary glucose quantities ranging from 60–120 g of glucose per day [30], underlining the critical role SGLT2 plays in the majority of glucose reabsorption in the proximal tubule. In contrast, SGLT1 loss-of-function mutations result in very little glucose excretion in the urine but manifest a significant glucose reabsorption defect in the intestine [30]. Due to the significant renal-specific role of SGLT2 in the kidney, it is a clear therapeutic target to alter glucose homeostasis as an approach for hyperglycemia and T2D.

## 3. Hyperglycemia, Type 2 Diabetes (T2D), and Urate

Metabolic disorders and disorders in insulin regulation/response can lead to dysfunction in circulating glucose levels and glucose storage, increasing plasma glucose levels above normal, resulting in the clinical pathology of hyperglycemia [31]. During hyperglycemia, glucose reabsorption by the proximal tubule increases from approximately 400 to 500 g per day to 500 to 600 g per day [29], a testament to the capacity of glucose reabsorption in the kidney and the strong evolutionary pressure to preserve every molecule of glucose, even in pathological states [32].

Hyperglycemia results in the upregulation of expression and function of both SGLT2 and SGLT1 to prevent the loss of the excess filtered glucose; however, this also preserves the underlying hyperglycemia [30]. The transport of glucose across the apical membrane is facilitated by sodium transport; thus, more glucose also results in significantly more sodium reabsorption in the early portion of the nephron, as well as additional water [30]. The reduced volume and sodium concentration of the tubular fluid is detected downstream at the macula densa in the thick ascending limb (TAL) [30]. The diminished sodium and fluid delivery are stimuli to increase the glomerular filtration rate (GFR) by tubuloglomerular feedback (TGF) to restore GFR, electrolyte, and fluid delivery [5,30]. Chronic hyperfiltration results from hyperglycemia and causes increased glomerular filtration pressure, leading to deleterious podocyte damage, proteinuria, and ultimately, diabetic nephropathy and diabetic kidney disease [5]. The pathological mechanisms of hyperglycemia on the proximal tubule have been studied extensively using model systems. Hyperfiltration at the glomerulus has been suggested, using mathematical modeling of the rat nephron, to also increase transporter density in the proximal tubule and is a flow-dependent process [33]. The in silico modeling was confirmed in the type 2 diabetes (db/db mice) mouse model, where hyperglycemia was found to significantly increase the expression of SGLT2 in the proximal tubule along with hypertrophy of the tubule, leading to increased sodium reabsorption [34], exacerbating the pathophysiological consequence of glucose hyperfiltration.

Hyperinsulinemia and insulin resistance often accompany sustained hyperglycemia [5]. Insulin and the insulin receptor pathways regulate a significant number of transporters in the proximal tubule, including SGLT2 in the S1/S2 and URAT1 in the S2/S3 segments [35,36]. In the context of hyperglycemia and the subsequent development of hyperinsulinemia, the sustained increase of insulin may drive increased urate reabsorption via the increased function of URAT1 [5]. An interventional trial tested this hypothesis by infusing insulin into healthy human subjects and found that this led to increased serum urate levels and worsened hyperuricemia [37,38]. Conversely, another important glucose homeostasis hormone, IGF-1, in in vitro transport studies, working via the IGF receptor, was found to stimulate urate transport by basolateral SLC2A9, OAT1/3, and apical ABCG2 but not the primary apical reabsorption transporter URAT1, suggesting IGF-1 may stimulate urate secretion [37,39]. These data convincingly link glucose homeostasis hormones and urate homeostasis; however, a causal pathological relationship between urate and glucose homeostasis or vice versa has been difficult to demonstrate.

Epidemiological studies have shown urate to be a significant independent risk factor for the development of metabolic disorders and disorders of glucose homeostasis. Several meta-analysis studies have shown that T2D patients have an increased risk of cerebral infarction and diabetic kidney disease in a dose-response manner, which increased 6% and 24% for every 1 mg/dL increase of serum uric acid, respectively [40–42]. A cohort study of Japanese adults followed for 5 years showed individuals with asymptomatic hyperuricemia (in the absence of diabetes and obesity, hypertension, CKD, or dyslipidemia) had a significantly increased incidence of hypertension, dyslipidemia, CKD, and being overweight/obese [43]. The increased incidence of developing T2D was significant in females but remained only a trend in males [43]. Subjects from the Rotterdam Study observed a similar sex difference, where serum urate levels in women were associated with prediabetes but not T2D, and the opposite was found for men [44]. These studies indicate that a clear sex difference exists between males and females when hyperuricemia is used as a predictor of future metabolic dysfunction. An additional study on women who had previous gestational diabetes mellitus (pGDM) found that urate levels were significantly higher in women with pGDM, which also correlated with BMI and carbohydrate disorders [45]. Multivariate analysis revealed a significant correlation of urate with BMI, creatinine levels, triglycerides, and family history of diabetes [45]. Women T2D patients with normal weight and normoglycemia also had higher urate levels, suggesting that T2D development is related to urate levels in women [45]. Serum urate levels are also positively associated in non-alcoholic fatty liver disease (NAFLD) prevalence. A cross-sectional study of Chinese patients with T2D found a progressive increased risk for NAFLD as serum urate levels increased [46], and serum urate levels were found to be positively associated with BMI, serum insulin, and lipid levels, which are common associated risk factors for NAFLD development [47]. These data support the idea that hyperuricemia is accompanied by general metabolic dysfunction, including high lipid and insulin levels and an increased BMI. However, like many studies, a direct causal link between hyperuricemia and T2D remains inconclusive.

In contrast to epidemiological studies, Mendelian randomization studies have provided a confusing picture of the relationship between hyperuricemia and T2D risk. Mendelian randomization uses genetic variants to evaluate the causality of a certain disease and eliminate confounding factors such as lifestyle and the environment. Mendelian randomization studies are a powerful tool but rely on a number of assumptions about the genetic instrumental variable, including that it produces no pleiotropic effects [48]. Given that we are still learning about the physiology of the urate transporters most often used, interpretation can be difficult. For example, in a Chinese population in which a causal relationship between serum urate levels and diabetes risk was studied, the genetic risk score was strongly associated with elevated urate levels and an increased risk for diabetes [49]. However, a study conducted on a European population using 24 loci associated with urate found that after a mean follow-up of 10 years, 43.6% of people developed T2D, but determined that urate levels do not increase the risk of T2D development [50]. And finally, a recent effort to study the relationship between insulin resistance and hyperuricemia determined that hyperinsulinemia leads to hyperuricemia but is not a result of it [51,52]. The role of urate in disease, as illuminated by Mendelian randomization studies, has been covered in an excellent recent review [53].

The definitive approach for testing a causal role for urate in metabolic disease is to reduce urate and observe alterations in either disease occurrence or progression using a randomized clinical trial (RCT). Significantly, recent large RCTs have empirically tested the hypothesis that lowering serum urate will have beneficial effects on cardiorenal disease. Both the CKD-FIX (Controlled Trial of Slowing of Kidney Disease Progression from the Inhibition of Xanthine Oxidase) and PERL (Preventing Early Renal Loss in Diabetes) trials tested whether the urate-lowering therapy (ULT) drug allopurinol was sufficient to prevent the decline of eGFR in patients with CKD (stages 3 and 4) or diabetic kidney disease (DKD) and T1D, respectively. Both studies found that lowering serum urate levels alone did not prevent eGFR decline in these patients [54,55]. Conversely, two different RCTs showed the opposite, where allopurinol intervention slowed the progression of CKD [56] by slightly increasing eGFR (+1.3 mL/min/1.73^2^), and patients with asymptomatic hyperuricemia and normal renal function saw an improvement in both endothelial dysfunction and eGFR [57,58]. Differences in the population characteristics and stage of CKD between studies may explain these conflicting results. Future RCTs that control for the underlying etiology of hyperuricemia could provide increased clarity.

Studies using animal models of hyperuricemia have been more uniform in their findings. Acute hyperuricemia mouse models, where the rapid onset of hyperuricemia is triggered by the chemical inhibition of the uricase enzyme by oxonic acid, have shown that hyperuricemia may induce insulin resistance, as indicated by both glucose and insulin tolerance testing and inhibition of IRS1 and AKT signaling [59]. A genetic mouse model of hyperuricemia, utilizing the knock in of the common human gout-causing ABCG2 variant Q141K, showed that a single amino acid substitution in ABCG2 caused not only hyperuricemia in male mice, but the mice also developed hyperinsulinemia, hyperglycemia, and showed evidence of increased fat deposition in the liver, supporting a causal role for either urate or ABCG2 dysfunction (or both) in glucose homeostasis [8,19,21,60,61]. Similar to several human cohort studies, a Uox KO mouse model generated by targeted deletion of 28 base pairs of the uricase oxidase gene found that male mice developed metabolic disorders involving impaired insulin secretion and were more susceptible to developing T2D, whereas females developed dyslipidemia and hypertension, emphasizing the sexual dimorphism of hyperuricemia between males and females [62]. In summary, mouse models of hyperuricemia more clearly support that hyperuricemia causes metabolic dysfunction, including insulin resistance and hyperinsulinemia, which may precede the development of T2D.

## 4. SGLT2 Inhibitors and Their Effects

Sodium glucose cotransporter 2 inhibitors (SGLT2is) are extremely effective, recently approved drugs for type 2 diabetes (T2D) used to promote glucosuria. The inhibition of SGLT2 (*SLC5A2*) reduces sodium-coupled glucose reabsorption in the early proximal tubule, and consequently, overwhelms the downstream glucose reabsorption capacity of SGLT1 (*SLC5A1*) [63]. The dominant role of SGLT2 in reabsorbing glucose has been validated in SGLT2 and SGLT1 knockout studies in mice, where SGLT2 was determined to be responsible for at least 97% of all glucose reabsorption located in the early proximal tubule, and the remaining 3% is scavenged by the late proximal tubule expressing SGLT1 [34]. The location of expression and physiological characteristics of SGLT2 and SGLT1 highlight the importance of pharmacologically targeting SGLT2 over SGLT1. By inhibiting SGLT2-mediated glucose reabsorption, the increased glucose transport and hypertrophy of the proximal tubule induced by hyperglycemia is mitigated.

The first SGLT inhibitor, phlorizin, was derived from the bark of an apple tree and was discovered to have a glucosiuric effect due to the dual inhibition of both SGLT2 and SGLT1 [64,65]. The structure of phlorizin is an O-glucoside (glucose ring connected to an oxygen) with two additional phenol rings attached to the oxygen atom, owing it a ten times higher affinity for SGLT2 than SGLT1 [65]. Although phlorizin was successful at normalizing plasma glucose concentrations and increasing insulin sensitivity and secretion in diabetic rats [66,67], its poor bioavailability and gastrointestinal side effects as a result of SGLT1 inhibition halted the progression of its potential use in humans. To overcome the short comings of dual SGLT inhibition, the pharmaceutical industry shifted to concentrating on synthesizing a more potent and selective SGLT2 inhibitor over SGLT1. The attention shifted to C-glucosides, which contain a heteroaromatic ring that rendered the molecule more metabolically stable than O-glucosides [68]. These efforts led to several second generation SGLT2 inhibitors approved for T2D in the US: canagliflozin, dapagliflozin, and empagliflozin.

### Clinical Benefits

Clinical trials for the three main SGLT2 inhibitors have characterized the clinical benefits of SGLT2 inhibitors: EMPA-REG OUTCOME (Empagliflozin Cardiovascular Outcomes and Mortality in Type 2 Diabetes), CANVAS (Canagliflozin Cardiovascular Assessment Study), and DECLARE-TIMI58 (Dapagliflozin effect on Cardiovascular Events). Together, all three trials showed that SGLT2 inhibition lead to many positive cardiorenal and metabolic effects, including lowering HbA1c levels, decreasing the risk of cardiovascular events, decreasing blood pressure, re-sensitization to insulin, weight loss, slowing the progression of diabetic kidney disease and CKD, and preserving glomerular filtration rate (GFR) [1,29,64]. SGLT2 inhibitors are considered insulin-independent drugs due to their effect in promoting glucosuria and natriuresis by the kidney instead of targeting metabolic tissues such as the pancreas, liver, and skeletal muscle. SGLT2 inhibitors are also beneficial to humans with T2D and high cardiovascular risk. The EMPA-REG OUTCOME clinical trial focused on addressing long-term effects of empagliflozin in those with GFR levels above 30 mL/min/1.73 m^2^ of body surface area, but included GFR ranges of 30–50 mL, 60–89 mL, and ≥90 mL/min/1.73 m^2^ [69]. In addition to reducing the development of nephropathy, empagliflozin treatment decreased the rate of hospitalization and death due to heart failure or cardiovascular disease [70].

The clinical trials for SGLT2is also revealed unexpected benefits. One side effect of pushing more sodium downstream with an SGLT2 blockade is the reduction in single nephron GFR that results in the macula densa/tubular glomerular feedback mechanism [30,71]. This reduction in GFR is beneficial in patients with early diabetic kidney disease and the coincident hyperfiltration. SGLT2 inhibition reduces GFR to pre-hyperglycemic levels and relieves pressure on the glomerulus, slowing the progression of diabetic nephropathy [5]. However, the observed drop in GFR appears to suggest that using SGLT2is for non-diabetic CKD patients might result in a dangerous drop in GFR. The recent EMPA-KIDNEY trial tested this very question [72]. The trial enrolled CKD patients with a GFR between 20 and 45 mL/min/1.73 m^2^, or CKD patients with an eGFR above 45 mL per min but with significant albuminuria [72]. The study unequivocally showed that for a wide range of CKD patients, SGLT2i lowered the risk of kidney disease progression or death from cardiovascular causes [72].

And finally, perhaps the most unexpected benefit of SGLT2 inhibitors is their direct effect of increasing renal urate excretion, resulting in significantly decreased serum urate levels through mechanisms that are poorly understood [73]. The EMPA, CANVAS, and DECLARE-TIMI58 trials collectively showed that the administration of SGLT2is was able to decrease serum urate to levels to those below clinically defined hyperuricemia of ≥6.0 mg/dL [70,74,75]. One of the interesting findings from the SGLT2i trials was the non-uniform effect size of SGLT2is on serum urate levels. The EMPEROR-REDUCED and DAPA-HF secondary analyses found that heart failure patients without diabetes or pre-diabetes showed a greater serum urate benefit from SGLT2i treatment than those with T2D [76,77]. A recent meta-analysis of SGLT2i randomized controlled trials, where serum urate levels were measured, found, through a subgroup analysis, that treatment with an SGLT2i had a smaller effect size on serum urate in CKD patients with an eGFR <60 mL/min/1.73^2^, and the longer the T2D duration, the smaller the SGLT2i effect on serum urate levels [78]. Why would T2D duration or eGFR reduction alter the effects of SGLT2is on serum urate levels and renal excretion? There are a number of possibilities. First, the inclusion/exclusion criteria between these studies are significantly different. The EMPEROR-REDUCED and DAPA-HF trials were primarily focused on heart failure patients, the Japanese CKD study focused on patients with CKD in the presence or absence of T2D, and the meta-analysis focused on serum urate levels with or without T2D [79]. The number of study participants and their ethnicities also greatly varied [76,79,80]. Clinically, heart failure patients and CKD patients with reduced eGFR are also mostly likely treated with medications to inhibit the RAAS system (i.e., ACE inhibitors) [55,81]. These medications decrease Na^+^ bicarbonate and water reabsorption in the proximal tubule, in turn affecting other sodium-driven reabsorption pathways including glucose and urate. These alterations may affect the mechanism underlying the SGLT2i role in renal urate excretion (see discussion below). Secondly, these trials may suggest proximal tubule injury from prolonged hyperglycemia present in T2D patients, and the resulting diabetic kidney disease (and eventually CKD with reduced eGFR) alters the urate transport mechanisms and the SGLT2i targets, reducing the effects of the SGLT2i to increase renal urate excretion. And finally, as the eGFR decreases, less urate is filtered, shrinking the potential pool of excreted urate, thus potentially obscuring the SGLT2i effect. In future clinical trials, it should be of upmost importance to address each comorbidity separately to effectively tease out which patient populations will benefit the most from SGLT2i treatment. These unanticipated but welcome observations of decreased serum urate levels with SGLT2i treatment illuminate the possibility for using SGLT2is in the treatment of hyperuricemia and gout.

## 5. SGLT2 Inhibitors and Gout

Patients with T2D and diabetic kidney disease/CKD often present with hyperuricemia and have a higher incidence of gout: 17% for all CKD patients and up to 33% for those with significantly reduced kidney function [82]. Several recent studies have assessed if the reduction in plasma urate levels with SGLT2is may confer protection from the development of gout. A longitudinal study comprised of approximately 300,000 adults with T2D measured the incidence of gout among these patients. It was found that those prescribed an SGLT2 inhibitor had a lower incidence (4.9 events per 1000 person) of developing gout compared to those treated with a GLP inhibitor (7.8 events per 1000 persons) [83]. A second similar retrospective study was performed using the UK biobank population data. The study utilized a baseline population characterized as having T2D and gout and then given either SGLT2is or comparators (e.g., GPL inhibitor). The study found that the gout patients given the SGLT2is had significantly fewer gout flares and significantly lower all causal mortality [7]. Both studies critically corroborated the link between tubular glucose and increased uric acid excretion by the kidney The potential benefits of SGLT2i for patients with gout has been recently reviewed [84].

## 6. How Do SGLT2 Inhibitors Lower Plasma Urate and Increase Uricosuria?

The idea of SGLT inhibition promoting renal urate excretion is not new, as the first nonspecific SGLT inhibitor, phlorizin, was observed to promote a uricosuric effect [85], but the mechanism underlying the effect remains controversial. The debate is important, because its resolution may reveal a critically important relationship between glucose reabsorption and urate homeostasis, providing new potential therapeutic targets for metabolic related diseases. Here, we discuss possible mechanisms of action.

The first possible mechanism of action is the off-target, nonspecific effects of SGLT2i drugs on urate transporter proteins, namely either URAT1 or SLC2A9. Competition between the inhibitor molecule and urate for transport would reduce reabsorption and thus increase the excretion of urate, resulting in the observed uricosuria. In vitro assays with the URAT1 or SLC2A9 proteins expressed in *Xenopus* oocytes showed that the SGLT2i Luseogliflozin had no effect on urate transport for URAT1 or SLC2A9 [73]. Further evidence against off-target drug effects of SGLT2is comes from mouse models. In mice, the genetic ablation of SGLT2 (*Slc5a2* knockout) increased renal urate excretion and increased the fractional excretion of urate [86]. And finally, patients with rare inherited mutations within the SGLT2 gene (*SLC5A2*; Familial Renal Glucosuria (FRG)), characterized by persistent glucosuria, with normal renal function and plasma glucose levels [87], also exhibit hyperuricosuria with hypouricemia, correlating again chronic high tubular glucose with increased uric acid excretion [88,89]. These data strongly reject a general nonspecific drug effect from SGLT2is on renal urate handling.

A second possibility is that the disruption of the bulk reabsorption of electrolytes and water in the early proximal tubule disrupts urate reabsorption. A recent study showed that SGLT2 inhibition also reduces the activity of the sodium proton exchanger 3, NHE3, in the early proximal tubule, disrupting Na^+^ bicarbonate reabsorption, resulting in a significantly greater delivery of Na^+^ downstream than predicted for the inhibition of SGLT2 alone [90]. This reduction in Na^+^ reabsorption in the early proximal tubule also prevents significant water reabsorption in the same segment, because water and Na^+^ are reabsorbed in an isotonic fashion. The loss of the fluid reabsorption would lower the relative tubular concentration of urate, possibly reducing the driving force for urate entry via the transcellular pathway (OAT4/10 and OAT1/3) in the early proximal tubule [8]. However, a pharmacological blockade of Na^+^ bicarbonate reabsorption in the proximal tubule with acetazolamide (via carbonic anhydrase inhibition) in patients produces a negligible increase in urinary urate excretion, suggesting that alterations in early proximal sodium and water movements alone do not directly affect urate handling in the nephron [91].

The third possible mechanism of action is based on the potential role of SLC2A9 to transport both urate and glucose. SLC2A9 has been described as a high-affinity, low-capacity glucose transporter, and therefore, increased tubular glucose resulting from the inhibition of SGLT2 in the early proximal tubule will shift significant amounts of glucose to the later proximal tubule, where it would compete with urate for SLC2A9-mediated transport, lowering the reabsorption of urate and increasing urinary urate excretion. Excellent work from an early study of both *SLC2A9* isoforms showed that SLC2A9-mediated urate transport was 45–60-fold faster than glucose transport [20], and critically, urate uptake was not affected by extracellular glucose up to 1mM. Urate and glucose do not compete for transport and may provide moderate levels of *trans*-stimulation [20,77,92]. The *trans*-stimulation of glucose may lead to increased urate secretion via apically localized SLC2A9 (the short isoform) in the collecting duct principal cells [64]. While an SLC2A9-mediated, glucose-dependent secretion mechanism is intriguing, in humans, the initial collecting duct localization [24] has not been confirmed and appears contrary to the reported gene expression of *SLC2A9* in the human nephron from single cell RNAseq [93,94]. Critically, direct empirical testing of the role of SLC2A9 through an SGLT2i-induced uricosuria mechanism was performed in mouse models with a genetic deletion of the key players, SGLT2 (*Slc5a2*), URAT1 (*Slc22a12*), and *SLC2A9*, with the data supporting a key role for URAT1 in SGLT2-mediated uricosuria, not SLC2A9 [86]. Furthermore, the deletion of SGLT1 enhanced the observed uricosuria, highlighting the role of the late proximal tubule, where URAT1 is primarily expressed, as the key mechanistic location [86]. Recently, a clinical trial was specifically designed to test for the key role of URAT1 in SGLT2i-mediated uricosuria in humans. The study, performed in patients with T2D and normal kidney function, showed that both a URAT1 inhibitor (benzbromarone) and Dapagliflozin produced an increase in urinary urate excretion; however, the combination of both therapies did not produce an additive effect; instead, the combined effect on urate excretion was the same as benzbromarone alone [6]. The combination of precise animal models and human clinical trials strongly supports a role for URAT1 in SGLT2i-mediated uricosuria.

**Figure 2.**
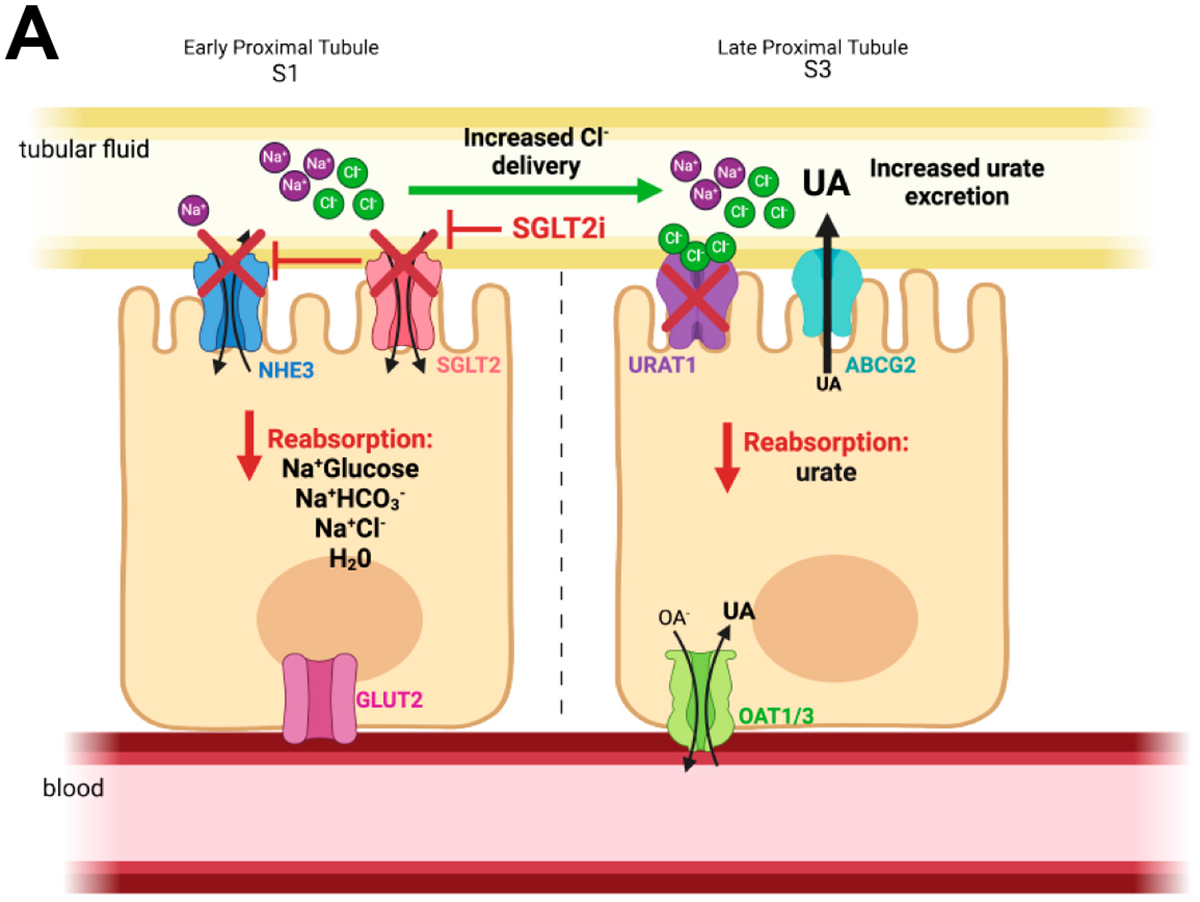

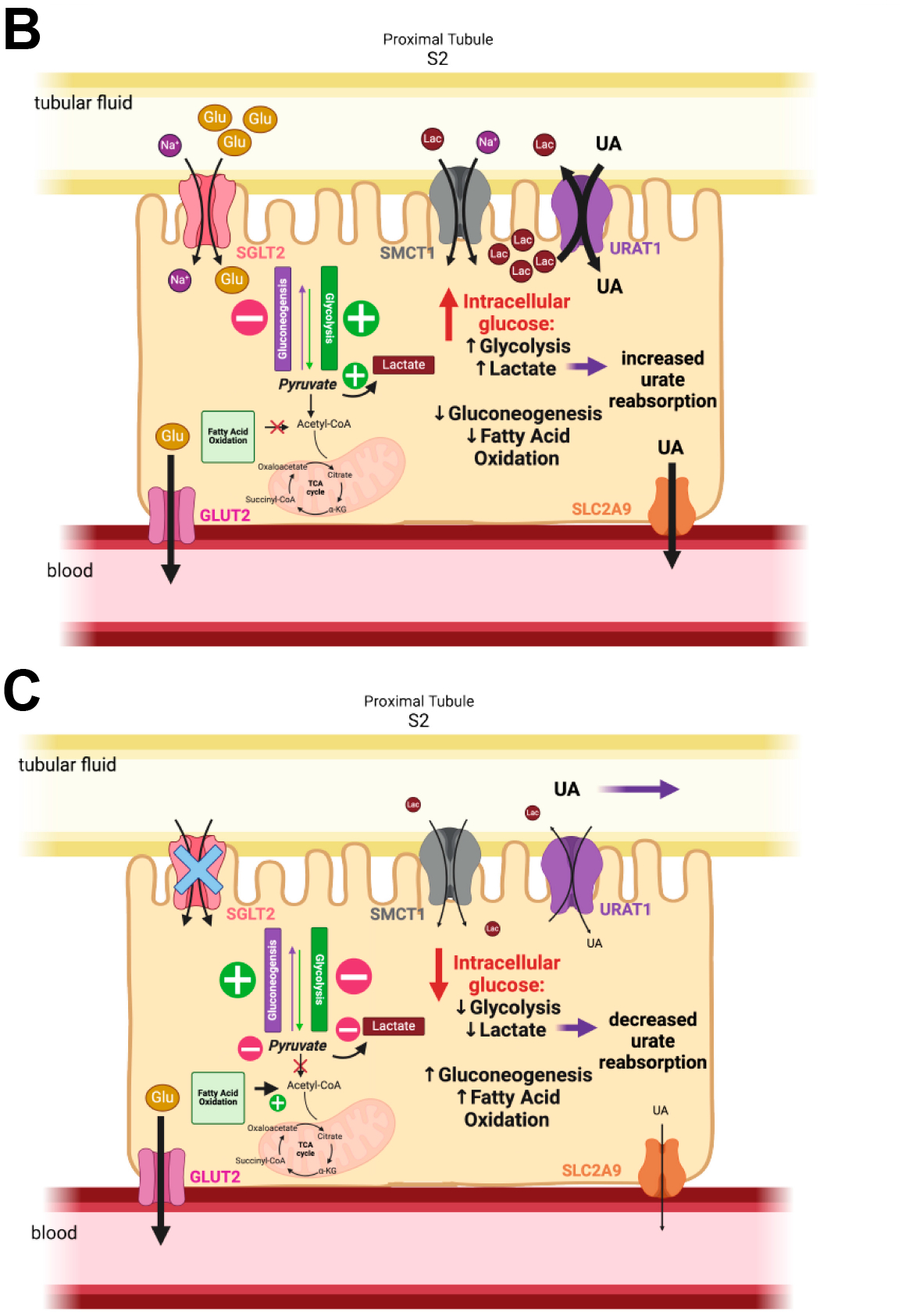
SGLT2i mediated uricosuria mechanisms of action. (**A**) Chloride delivery hypothesis: Reabsorption of NaCl, Na–glucose, and NaHCO_3_^−^ is inhibited by SGLT2i treatment in the early proximal tubule (S1) through the apical transporters SGLT2 and NHE3. As a result, sodium and chloride ions in the tubular fluid accumulate and are delivered to the downstream S3 segment, where apical URAT1 and ABCG2, the reabsorptive urate and secretory transporters are located. The increased delivery of chloride ions effectively inhibits URAT1 transporter activity, preventing the reabsorption of tubular urate, and thus, increasing urinary urate excretion by the kidneys. (**B**,**C**) Metabolism hypothesis: In the normal functioning proximal tubule, gluconeogenesis is energetically favored for cellular ATP production over glycolysis, where glucose is made by the proximal tubule cell instead of being used as the substrate for ATP production. (**B**) During the state of hyperglycemia, increased transporter activity of SGLT2 leads to increased sodium and glucose reabsorption from the tubular fluid. As a result, the excess glucose is used for glycolysis for pyruvate production and ultimately, Acetyl-CoA and entry into the TCA cycle, replacing fatty acid oxidation (FAO)-generated Acetyl-CoA. The increase in pyruvate production results in significant increases in intracellular lactate concentrations. The excess lactate also enters the tubular fluid, where the apical Na^+^/lactate cotransporter SMCT1 is expressed. As a result, more sodium and lactate are reabsorbed back into the cell, sustaining high intracellular lactate levels and *trans*-stimulating the reabsorption of urate via URAT1, resulting in a pathological positive feedback loop. (**C**) SGLT2 inhibition by an SGLT2i prevents the excess reabsorption of glucose, reducing glycolysis and stimulating gluconeogenesis, together significantly suppressing intracellular lactate. As a result of the decreased intracellular lactate levels, the *trans*-stimulation of URAT1 and urate reabsorption is significantly diminished, and thus, increases urinary urate excretion. Figures were created using Biorender.

How then do URAT1 and SGLT2 functionally interact? As noted above, SGLT2is acutely inhibit both SGLT2 and NHE3 activities, though the mechanism of NHE3 inhibition is not yet clear, resulting in significantly less absorption of Na^+^, Cl^*−*^, bicarbonate, glucose, and water in the early proximal tubule [95]. The delivery of NaCl to macula densa cells triggers the downregulation of single nephron GFR via TGF [5]. The Na^+^ and water delivery to the distal nephron results in a natriuresis and diuresis [95]. And finally, there is significant glycosuria and uricosuria. Of these acute consequences of SGLT2 inhibition, the delivery of more Cl^*−*^ to the late proximal tubule may be the most significant. Micro-puncture studies studying the inhibition of SGLT2 and NHE3 demonstrated the enhanced delivery of Cl^*−*^ to the downstream segments [71], in part as a result of the dilution of the transepithelial Cl^−^ gradient and persistent tubular Na^+^, both working to reduce paracellular Cl^−^ reabsorption in the S2–S3 segments. URAT1 is extremely sensitive to extracellular Cl^−^ concentrations [77,96], and in vitro studies showed that the removal of Cl^−^ from the bathing solution resulted in a 17–21-fold increase in urate influx [77]. Thus, the relative concentration of tubular Cl^−^ in the late proximal tubule may have a significant effect on the reabsorption of urate via URAT1 (Figure 2A). Recent longitudinal studies have looked at the chronic effects of SGLT2is on human physiology and have shown that within two weeks, there is upregulation of Na^+^, Cl^−^, bicarbonate, and water reabsorption in the later nephron segments, and the acute natriuresis and diuresis disappears [95]. Interestingly, the glucosuria and uricosuria persist, as does increased fractional excretion for lithium, an indicator that the proximal tubule is not compensating or becoming resistant to the drugs [95]. The persistent increase in Cl^−^ delivery to the latter portions of the proximal tubule may be responsible for a persistent blockade of URAT1-mediated urate reabsorption and the resulting uricosuria.

A second intriguing, possible mechanism of functional interaction of SGLT2 and URAT1 implicates a shift in renal epithelial cell metabolism and intracellular lactate levels. Hyperglycemia and the increased glucose reabsorption via SGLT2 shift the proximal tubule epithelial cell metabolism away from fatty acid oxidation (FAO)-fueled oxidative phosphorylation (OXPHOS) toward increased glycolysis and pyruvate generation used for OXPHOS [29] (Figure 2B). Increased proximal tubule glycolysis and reduced gluconeogenesis also result in an increased production of lactate [29,97]. The increase of intracellular lactate in the S2 proximal tubule results in additional *trans*-stimulation of URAT1-mediated urate reabsorption [15,77] (Figure 2A). The inhibition or genetic ablation of SGLT2 function may reverse these processes: (1) reduced glucose reabsorption shifts proximal tubule cellular metabolism back to FAO-based ATP generation and reduced glycolysis [29]; (2) the reduction of glucose reabsorption also activates starvation pathways and gluconeogenesis [29], primarily using lactate as the fuel, further lowering intracellular and luminal lactate levels [97]; (3) reduced intracellular lactate may then prevent the *trans*-stimulation of URAT1-mediated urate reabsorption, leading to significantly higher levels of urinary urate excretion (Figure 2C). The hypothesis for both a role for altered Cl^*−*^ delivery or proximal tubule cell metabolism in SGLT2i-induced uricosuria remain untested and represent a potential future area of interest for furthering our understanding of the relationship between glucose and urate handling in the proximal tubule.

## 7. Conclusions

The unanticipated effect of SGLT2 inhibitor treatment on serum urate levels strongly suggests that renal handling mechanisms of glucose and uric acid are intimately intertwined, and those with hyperuricemia and gout may benefit from SGLT2i treatment in combination with existing ULT strategies. Several debates in the field remain, including the renal mechanism(s) responsible for the observed beneficial effect of uricosuria by SGLT2 inhibition and whether T2D causes hyperuricemia or vice versa. Current, ongoing, and future studies of SGLT2is and uric acid transport mechanisms will hopefully resolve these highly debated questions in the near future.
